# The changing contributory role to infections of work, public transport, shopping, hospitality and leisure activities throughout the SARS-CoV-2 pandemic in England and Wales

**DOI:** 10.3310/nihropenres.13443.1

**Published:** 2023-11-03

**Authors:** Susan Hoskins, Sarah Beale, Vincent G Nguyen, Thomas Byrne, Alexei Yavlinsky, Jana Kovar, Erica Wing Lam Fong, Cyril Geismar, Annalan M. D. Navaratnam, Martie van Tongeren, Anne M. Johnson, Robert W. Aldridge, Andrew Hayward

**Affiliations:** 1 Institute of Health Informatics, University College London, London, England, NW1 2DA, UK; 2 Institute of Epidemiology and Health Care, University College London, London, WC1E 6BT, UK; 3 Department of Infectious Disease Epidemiology, Imperial College London, London, W2 1NY, UK; 4 Centre for Occupational and Environmental Health, The University of Manchester, Manchester, England, UK; 5 Institute for Global Health, University College London, London, England, WC1E 6BT, UK

**Keywords:** SARS-CoV-2, social-activities, work, transport, hospitality, leisure

## Abstract

**Background:**

Understanding how non-household activities contributed to severe acute respiratory syndrome coronavirus 2 (SARS-CoV-2) infections under different levels of national health restrictions is vital.

**Methods:**

Among adult Virus Watch participants in England and Wales, we used multivariable logistic regressions and adjusted-weighted population attributable fractions (aPAF) assessing the contribution of work, public transport, shopping, and hospitality and leisure activities to infections.

**Results:**

Under restrictions, among 17,256 participants (502 infections), work [adjusted odds ratio (aOR) 2.01 (1.65–2.44), (aPAF) 30% (22–38%)] and transport [(aOR 1.15 (0.94–1.40), aPAF 5% (-3–12%)], were risk factors for SARS-CoV-2 but shopping, hospitality and leisure were not. Following the lifting of restrictions, among 11,413 participants (493 infections), work [(aOR 1.35 (1.11–1.64), aPAF 17% (6–26%)] and transport [(aOR 1.27 (1.04–1.57), aPAF 12% (2–22%)] contributed most, with indoor hospitality [(aOR 1.21 (0.98–1.48), aPAF 7% (-1–15%)] and leisure [(aOR 1.24 (1.02–1.51), aPAF 10% (1–18%)] increasing. During the Omicron variant, with individuals more socially engaged, among 11,964 participants (2335 infections), work [(aOR 1.28 (1.16–1.41), aPAF (11% (7–15%)] and transport [(aOR 1.16 (1.04–1.28), aPAF 6% (2–9%)] remained important but indoor hospitality [(aOR 1.43 (1.26–1.62), aPAF 20% (13–26%)] and leisure [(aOR 1.35 (1.22–1.48), aPAF 10% (7–14%)] dominated.

**Conclusions:**

Work and public transport were important to transmissions throughout the pandemic with hospitality and leisure’s contribution increasing as restrictions were lifted, highlighting the importance of restricting leisure and hospitality alongside advising working from home, when facing a highly infectious and virulent respiratory infection.

## Plain Language summary

Establishing which activities and venues that were restricted in England and Wales during lockdowns were the most likely to lead to severe acute respiratory syndrome coronavirus 2 (SARS-CoV-2) infections will help us understand how useful the restrictions were and will help us to develop proportional responses to future public health threats. We found that during periods of intense restrictions (October 2020–May 2021) many people became infected with SARS-CoV-2 if they left home to go to work or used public transport. During the period after most public health restrictions were lifted (September–mid-December 2021), while many people continued to become infected at work or if they used public transport, indoor hospitality and indoor leisure venues became increasingly important as places where people became infected. During the Omicron wave of the pandemic (December 2021–April 2022), by which point there were very few restrictions on people’s activities and many people were visiting hospitality and leisure venues with increasing frequency, people continued to become infected at work and on public transport, but hospitality and leisure venues were nearly as important places where people became infected. As essential activities led to most cases during periods of tight restrictions and leisure and hospitality activities became increasingly important under periods when rules were more relaxed, it is important to recognise how vital it was to encourage people to work from home, reduce public transport use and restrict visits to leisure and hospitality settings when the country was faced with a fast-spreading virus which killed many people. Outdoor use of leisure and hospitality venues appeared to be safer than indoor use.

## Introduction

Prior to the availability of effective vaccines, repeated ‘lockdowns’ were a critical element of the coronavirus disease 2019 (COVID-19) pandemic response in the UK to reduce transmission of severe acute respiratory syndrome coronavirus 2 (SARS-CoV-2) and resulting hospitalisation and mortality ^1, 2^. Lockdowns compelled the public to stay at home with advice to work from home where possible and minimise public transport use, and a range of retail, hospitality and leisure venues were closed, restricting non-essential activities and social mixing ^1, 2^. After the national vaccination programme began in England and Wales and public access to free testing increased, restrictions were gradually relaxed by increasing the range of public venues that could be visited and reducing restrictions on the number of people outside the home who could be visited ^1– 3^. The majority of activity and social mixing restrictions were removed in July 2021 (in England) and August 2021 (in Wales) ^2, 4^. While evidence suggests that restriction measures substantially reduced SARS-CoV-2 transmission and associated outcomes, these measures have far-reaching financial, social, and health-related impacts ^5– 7^. Understanding the transmission risk associated with different activities and venues affected by restriction measures is crucial to develop an understanding of the effectiveness of these measures and to develop proportional responses to future public health threats. 

Several studies from varying regions and phases of the pandemic have aimed to investigate SARS-CoV-2 infection risk associated with different activities. Identified risk factors during the first wave of the pandemic include having an increased number of non-household contacts, air travel, employment, shopping, frequency of attending events of at least 10 people, participating in more than one non-essential activity per day, attending various indoor settings including restaurant visits, places of worship, gyms or salons ^8– 10^. However, first-wave data were limited as global testing capacity was low and infections data were likely incomplete. During periods when restrictions were lifted and public venues starting to reopen, data are conflicting. Public transport use has been associated with increased odds of infection, as has shopping at convenience stores and visiting a place of worship ^11, 12^. The drinking of alcohol in restaurants or bars and attending events with singing, or attending bars, parties of private ceremonies have been associated with greater odds of infection ^12, 13^. Visiting indoor leisure venues including fitness centres has also been found to increase infection risk, while working remotely has been found to reduce infection odds ^13, 14^. However, conflicting data from that period suggest that there is no relationship with infection for a range of essential and non-essential activities or public transport use, shopping, or leisure activities ^12, 14^. For a period, some months after the removal of most national restrictions, drinking in bars and restaurants and visiting fitness centres has been found to be associated with odds of infection ^15^.

Differences in which activities are associated with increased odds of infection may reflect different rates of infection and the implementation of other non-pharmaceutical interventions across regions, as well as differences in study design and collection of activity data. The impact of activities on SARS-CoV-2 transmission is likely to be differential depending on levels of national public health restrictions, as well as features of the dominant variant at the time of investigation and rates of infection and contact in the population. Comparison of the relative contribution of activities to transmission across different pandemic periods with comprehensive adjustment for potential confounding is lacking, particularly for later phases of the pandemic following the emergence of the highly infectious Omicron variant. This is warranted to inform responses to future public health emergencies. 

To address this research gap, we used data from the Virus Watch Community Cohort Study in England and Wales, a study which collects individual level data both on infections and activities across time, to investigate how work, public transport, shopping, and hospitality and leisure activities contributed to SARS-CoV-2 infections during periods with different levels of national public health restrictions. 

## Methods

The current study was embedded within the Virus Watch community cohort study, which has been active since June 2020 and involves households across England and Wales completing a detailed baseline survey related to demographic and clinical characteristics, then subsequent weekly surveys about acute illnesses, COVID-19 vaccination, and SARS-CoV-2 testing (PCR or lateral flow) and monthly surveys about socio-demographic and behavioural topics ( *e.g.,* activity patterns). Linkage was also performed to national records of SARS-CoV-2 testing and vaccination. The Virus Watch cohort methodology has been described in detail elsewhere ^16^.

Within this cohort, we examined the contributory role of non-household activities to SARS-CoV-2 infections during three periods of differing levels of public health restrictions in England and Wales: a period under intense restrictions and during the second wave of the UK pandemic (October 2020–May 2021); a period immediately following the lifting of national public health restrictions on 19th July 2021 during the third wave of the UK pandemic dominated by the Delta variant (September–mid-December 2021); and a period characterised by no restrictions for the majority of the time (with the exception of a return to mask guidance and self-testing prior to visiting vulnerable friends in the festive period), a level of social activity engagement closer to pre-pandemic levels, and dominated by the highly transmissible Omicron variant (December 2021–April 2022). 

### Patient and Public Involvement

Patients and/or the public were not involved in the development or dissemination of the current study. Due to Virus Watch being an urgent public health study during the COVID-19 pandemic, patients and/or the public were not involved in its initial design. An advisory group comprising members of the public, community leaders, charities and policy organisations provided feedback into the recruitment of ethnic minority participants and health equity focused analyses within Virus Watch but were not directly involved in the design or dissemination of this study.

### Ethics approval

This study was performed in line with the principles of the Declaration of Helsinki. Approval was granted by the Hampstead NHS Health Research Authority Ethics Committee, with the ethics approval number - 20/HRA/2320. Informed written consent was obtained from all individual participants included in the study.

### Study participants

For each period, within the Virus Watch community study we identified a cohort of adult participant aged 18 years and above who completed monthly activity surveys. Infections were included if testing PCR or lateral flow positive during the relevant period, unless there was evidence of recent infection in the previous three months, as a positive test may have signalled ongoing infection acquired prior to the study period. 

For the period under intense restrictions, we identified adult participants answering two monthly activity surveys during the periods 1/12/2020–10/12/2020 and 17/02/2021–28/02/2021. Virus Watch activity surveys commenced in November 2020, so activity data were not available for earlier in the pandemic wave and we did not include responses from the early January 2021 activity survey as activities during the Christmas holiday period (measured in this survey) were not considered representative for the broader period under consideration. Infections were included if testing PCR or lateral flow positive between 01/10/2020 and 01/05/2021 unless there was evidence of recent infection in the previous three months.

For the period following the lifting of national public health restrictions, we identified adults who completed the three activity surveys during the periods 22/09/2021–29/09/2021, 19/10/2021–26/10/2021 and 16/11/2021–23/11/2021. We did not include responses from the August survey as many participants were on holiday and survey completion rates were low. Infections were included if testing PCR or lateral flow positive between 01/09/2021 and 16/12/2021 unless there was evidence of recent infection in the previous three months. 

For the final period, characterised by the wide-spread circulation of the Omicron variant, we identified adult participants answering activity surveys during the periods 05/01/2022–12/01/2022, 15/02/2022–22/02/2022, and 23/03/2022–30/03/2022. Infections were included if testing PCR or lateral flow positive between 11/12/2021 and 30/03/2022 unless there was evidence of recent infection in the previous three months.

Some data from periods of intense restrictions and following the lifting of national public health restrictions have been used elsewhere, but the present investigation included more identified cases and further adjustment for potential confounding as well as further data from the Omicron wave of infections ^17, 18^.

### Outcome variable

Participants were considered to have had a SARS-CoV-2 infection if they had a positive PCR or lateral flow test identified through self-report or linkage to national records of SARS-CoV-2 testing (Public Health England's Second Generation Surveillance System (SGSS) including infections identified through hospitalisations and community testing). The linkage was performed by NHS Digital. Self-reported and linked tests were matched and, while there was a high degree of overlap, linkage was used as a preferred source due to accuracy.

### Exposure variables

In the Virus Watch monthly surveys, participants reported the number of days that they engaged in a range of activities in week preceding each survey and their estimated number of close contacts. Using these surveys, we examined the frequency of the following activities: attending work or education outside the home, using public or shared transport, visiting retail settings, and visiting indoor and outdoor hospitality or leisure settings. 

For each period, we averaged the frequency of each activity and the number of close contacts across all relevant surveys to give an estimated overall frequency of the activity during each period. We created the following composite variables: public transport activities (combining use of taxi, bus, over and underground rail and air travel), retail activities (combining use of essential and non-essential shops) and indoor hospitality (eating in an indoor restaurant, cafe or canteen; going to an indoor bar, pub or club; and going to an indoor party), outdoor hospitality (eating in an outdoor restaurant, cafe or canteen; going to an outdoor bar, pub or club; and going to an outdoor party), indoor leisure (attending a gym, the theatre, the cinema, a concert or sports event), outdoor leisure (outdoor team sport), and non-social activities (visiting barber, hairdresser, beautician or nail salon). For the period under national restrictions, when visits to hospitality and leisure venues was largely curtailed through closures, we created a composite variable for ‘any other non-work non-public transport non-retail activity’ to include visiting a canteen/café or restaurant, a bar, pub, club or disco, an indoor gym or outdoor team sport, attending a party, visiting a sports event, concert, cinema, or theatre, a hairdresser, barber or nail salon or a place of worship. As the Virus Watch surveys were adapted throughout the pandemic waves, the earlier surveys during times of lockdown restrictions gathered information on indoor and outdoor activities as combined groups whereas the latter surveys disaggregated the activities. Retail was categorised at levels that were appropriate for each time period. 

We conducted univariate analyses to compare the proportion of infected participants based on their weekly frequency of going to work, the composite measures, and each exposure individually. We also conducted multivariate logistic regression using the following adjustment set, which was identified using a directed acyclic graph (DAG), to estimate the direct effect of each non-household activity exposure: mutual adjustment for each activity, sex, region, living arrangements (alone or with children), residence in a deprived area (utilizing a combination of rural, urban, conurbation area of residence, and deprivation status), and vaccine status. Age was not included in the adjustment set indicated by the DAG due to its effect operating through included variables; however, we performed a sensitivity analysis including age due as it widely regarded as a confounder for behavioural activities.

The timing of the UK vaccine policies influenced how we categorised vaccine status. As few participants had access to vaccines prior to our restriction period cohort, vaccine status was determined if a participant received any vaccine during the cohort period. During the second period, following the lifting of restrictions, only 1% of the cohort received a vaccine, as the vast majority had been vaccinated by July 2021 and the booster programme largely drive by the arrival of the Omicron variant had not started. For this period, therefore, we categorised vaccine status based on having none or at least one vaccine at the start of the wave. For the final period, we again categorised vaccine status based on being vaccinated or not during the study period. 

We used inverse probability weighting to account for the older age structure of Virus Watch monthly respondents and calculated age-weighted, adjusted multivariate population attributable fractions (aPAF) to estimate the impact of each exposure on non-household transmission in the cohort. Weights were derived from estimates of the age structure of the UK population ^19^. Missing data were sparse, and all observations were included in the univariate analyses, while complete case analysis performed for the multivariate adjusted models and resulting PAFs. Analyses were conducted in STATA version 16. 

## Results

### Cohort characteristics

During the period under restrictions, among 17,256 participants, 502 cases were identified ( Table 1). During the period immediately following the lifting of public health restrictions, among 11,413 participants 493 cases were identified. During the highly infectious Omicron wave as individuals engaged more frequently in social activities, among 11,964 participants, we identified 2335 infections. The participants in the cohorts who answered the Virus Watch activity surveys across the waves were similar in terms of demographics. Participants were in majority female (around 57%), and lived with another person (around 75%), few lived with children, though the proportion living with children was double during the earliest period under restrictions (12%) that of the later periods (6%). Just under half of participants (46%) lived in an urban area and most participants (around 58%) lived in postcodes classified as low deprivation according to the UK Office for National Statistics. The participants answering the surveys during the earliest wave were slightly younger (55% of working age) than those answering during the period immediately following the lifting of restrictions (48% working age) or during the Omicron wave (46% of working age). The cohorts were largely vaccinated. 

**Table 1.** Characteristics of participants during three periods of the SARS-CoV-2 pandemic in England and Wales.

		Restriction period			Post lifting of national restrictions			Omicron wave		
Characteristic	Category	N=17,256 (% in category)	Number of infections n= 502 (% within category)	Unadjusted Odds ratio, 95% confidence interval, p- value	N=11,413 (% in category)	Number of infections n= 493 (% within category)	Unadjusted Odds ratio, 95% confidence interval, p- value	N=11, 964 (% in category)	Number of infections n= 2,335 (% within category)	Unadjusted Odds Ratio, 95% confidence interval, p- value
Sex	Male Female Missing	7555 (44%) 9519 (56%) 182	191 (2.53%) 307 (3.23%)	1.00 1.28 (1.06 – 1.54) P=0.0069	4857 (43%) 6470 (57%) 86	209 (4.3%) 278 (4.3%)	1.00 0.99 (0.83 – 1.19) P=0.9869	5085(43%) 6857 (57%) 22	995 (19.57%) 1334 (19.45%)	1.00 0.99 (0.91 – 1.09) P=0.8778
Age	18 – 34 35 – 49 50 – 64 65 – 79 80 plus	1322 (8%) 2332 (14%) 5812 (34%) 7296 (42%) 494 (3%)	66 (4.99%) 111 (4.76%) 178 (3.06%) 141 (1.93%) 6 (1.21%)	1.00 0.95 (0.69 – 1.29) 0.60 (0.45 – 0.80) 0.38 (0.28 – 0.51) 0.23 (0.10 – 0.54) P<0.0001	467 (4%) 1019 (9%) 3884 (34%) 5683 (49%) 359 (3%)	35 (7.5%) 92 (9.0%) 204 (5.3%) 153 (2.7%) 9 (2.5%)	1.22 (0.82 – 1.84) 0.68 (0.47 – 0.99) 0.34 (0.23 – 0.49) 0.32 (0.15 – 0.67) 1.00 P<0.0001	389 (3.25%) 1013 (8.47%) 4075 (34.06%) 6127 (51.21%) 360 (3.01%)	119 (30.59%) 272 (26.85%) 825 (20.25%) 1,080 (17.63%) 39 (10.83%)	1.00 0.83 (0.64 – 1.08) 0.58 (0.46 – 0.72) 0.49 (0.39 – 0.61) 0.28 (0.19 – 0.41) P<0.0001
Vaccination status	No Yes	1,298 (8%) 15,958 (92%) *during wave	64 (4.93%) 438 (2.74%)	1.00 0.54 (0.42 – 0.71) P<0.0001	687 (6%) 10,726 (94%) *by start of period	29 (4.3%) 464 (4.2%)	1.00 1.03 (0.69 – 1.51) P=0.8955	9,891 (83%) 2,073 (17%) *during wave	1,968 (19.90%) 367 (17.70%)	1.00 0.87 (0.77 – 0.98) P=0.0207
Region	East Midlands East of England London North East North West South East South West Wales W. Midlands Yorkshire & The Humber Missing	1652 (10%) 3598 (21%) 1935 (11%) 906 (5%) 1967 (12%) 3109 (18%) 1445 (8%) 432 (3%) 1030 (6%) 957 (6%) 225	49 (2.97%) 113 (3.14%) 91 (4.70%) 29 (3.20%) 59 (3.00%) 58 (1.87%) 33 (2.28%) 8 (1.85%) 24 (2.33%) 36 (3.76%)	1.00 1.06 (0.75 – 1.49) 1.61 (1.13 – 2.29) 1.08 (0.68 – 1.72) 1.01 (0.69 – 1.49_ 0.62 (0.42 – 0.91) 0.76 (0.49 – 1.19) 0.62 (0.29 – 1.31) 0.78 (0.48 – 1.28) 1.28 (0.83 – 1.98) P<0.0001	1065 (9%) 2551 (23%) 1202 (11%) 581 (5%) 1192 (11%) 2210 (19%) 924 (8%) 266 (2%) 626 (6%) 615 (5%) 181	35 (3.3%) 92 (3.6%) 57 (4.7%) 35 (6.0%) 62 (5.2%) 102 (4.6%) 33 (3.6%) 18 (6.8%) 33 (5.3%) 15 (2.4%)	1.00 1.10 (0.74 – 1.64) 1.47 (0.95 – 2.25) 1.89 (1.17 – 3.05) 1.61 (1.06 – 2.46) 1.42 (0.96 – 2.11) 1.09 (0.67 – 1.77) 2.14 (1.19 – 3.83) 1.64 (1.01 – 2.66) 0.74 (0.39 – 1.36)	1142 (10%) 2443 (21%) 1139 (10%) 590 (5%) 1244 (11%) 2407 (20%) 1079 (9%) 361 (3%) 684 (6%) 714 (6%) 161	215 (18.83%) 492 (20.14%) 245 (21.51%) 125 (21.19%) 248 (19.94%) 480 (19.94%) 204 (18.91%) 52 (14.40%) 126 (18.42%) 120 (16.81%)	1.00 1.09 (0.91 – 1.29) 1.18 (0.96 – 1.45) 1.16 (0.91 – 1.48) 1.07 (0.88 – 1.32) 1.07 (0.89 – 1.28) 1.01 (0.81 – 1.24) 0.73 (0.52 – 1.01) 0.97 (0.76 – 1.24) 0.87 (0.68 – 1.11) P=0.0687
Lives alone	Lives alone Lives with someone Missing	3570 (21%) 13,667 (79%) 19	110 (3.08%) 392 (2.87%)	1.00 0.93 (0.75 – 1.15) P=0.5032	2,869 (25%) 8,544 (75%)	81 (2.8%) 412 (4.8%)	1.00 1.74 (1.37 – 2.22) P<0.0001	3124 (26.11%) 8840 (73.89%)	518 (16.58%) 1817 (20.55%)	1.00 1.30 (1.17 -1.45) P<0.0001
Lives with children	No Yes Missing	15,167 (88%) 2070 (12%) 19	405 (2.67%) 97 (4.69%)	1.00 1.79 (1.43 – 2.25) P<0.0001	10,679 (94%) 734 (6%)	404 (3.8%) 89 (12.1%)	1.00 3.51 (2.57 – 4.47) P<0.0001	11,267 (94%) 697 (6%)	2128 (18.89%) 207 (29.70%)	1.00 1.81 (1.53 – 2.15) P<0.0001
Residential area	Any rural Any urban Any conurbation missing	4533 (27%) 7887 (46%) 4591 (27%) 245	105 (2.32%) 205 (2.60%) 190 (4.14%)	1.00 1.13 (0.89 – 1.43) 1.82 (1.43 – 2.32) P<0.0001	2886 (26%) 5209 (46%) 3137 (28%) 181	106 (3.7%) 216 (4.2%) 160 (5.1%)	1.00 1.13 (0.89 – 1.43) 1.41 (1.09 – 1.81) P=0.0201	3153 (27%) 5570 (47%) 3080 (26%) 161	549 (17.41%) 1094 (19.64%) 664 (21.56%)	1.00 1.16 (1.04 – 1.29) 1.30 (1.15 – 1.48) P=0.0002
Deprivation score (IMD quintile) 1= most deprived	1 2 3 4 5 Missing	1333 (8%) 2535 (15%) 3479 (20%) 4538 (27%) 5146 (30%)	61 (4.58%) 99 (3.91%) 114 (3.28%) 108 (2.38%) 118 (2.29%)	2.04 (1.49 – 2.80) 1.73 (1.32 – 2.27) 1.44 (1.11 – 1.87) 1.04 (0.79 – 1.35) 1.00 P<0.0001	852 (8%) 1589 (14%) 2,290 (20%) 3,032 (27%) 3,469 (31%) 181	44 (5.2%) 69 (4.3%) 94 (4.1%) 141 (4.7%) 134 (3.9%)	1.36 (0.96 – 1.92) 1.13 (0.84 – 1.52) 1.07 (0.81 – 1.39) 1.21 (0.95 – 1.55) 1.00 P=0.3764	825 (7%) 1613 (14%) 2366 (20%) 3090 (26%) 3909 (33%) 161	184 (22.30%) 317 (19.65%) 481 (20.33%) 564 (18.25%) 761 (19.47%)	1.19 (0.99 – 1.42) 1.01 (0.87 – 1.17) 1.06 (0.93 – 1.19) 0.92 (0.82 – 1.04) 1.00 P=0.0865

### Participant behaviour

During the period under restrictions, under one-third (29%) of participants left home for work or education, with this proportion increasing to around 35% during both the period following the lifting of restrictions and during the Omicron period ( Table 2). During the period under national restrictions, a similar proportion (28%) of participants to those leaving home for work used any form of public transport, however this proportion increased to nearly half of the cohort (48%) during the period after the lifting of restrictions and remained at 42% of the cohort during the Omicron wave. During the period under restrictions, 43% of participants did three or more non-household activities a week ( Table 2), although the proportions engaging in any single non-work non-transport non-retail activity was never more than 4% ( Table 3). Once restrictions were lifted, nearly half of participants were visiting indoor (43%) or outdoor (41%) hospitality or indoor leisure (41%) venues and more than one-third (35%) undertook non-social activities including visiting a hairdresser, beautician or nail salon. After the restrictions had been lifted for some months, during the Omicron wave, the proportion of participants visiting indoor hospitality venues was nearly double (77%) that of the period immediately following the lifting of restrictions, while the proportion visiting hospitality venues outdoors (30%) dropped slightly. The proportion of participants visiting indoor leisure venues (39%), outdoor leisure (7%) and non-social activities (35%) stayed the same during the Omicron period as the period immediately following the lifting of restrictions.

**Table 2.** Unadjusted infection odds associated with activities during three periods of the SARS-CoV-2 pandemic.

		Restriction period			Post lifting of national restrictions			Omicron wave		
Activity	Weekly frequency	Number (%) in cohort (17,258)	Number (%) of cases (n=502)	Odds Ratio (95% CI), p	N=11,413 (% in category)	Number of infections n= 493 (% within category)	Unadjusted odds ratio (95% CI), p	N=11, 964 (% in category)	Number of infections n= 2,335 (% within category)	Unadjusted odds ratio (95% CI), p
Leaving home for work or education	No Yes	12,228 (71%) 5028 (29%)	266 (2.18%) 236 (4.69%)	1.00 2.21 (1.85 – 2.65) p<0.0001	7294 (64%) 4119 (36%)	259 (3.6%) 234 (5.7%)	1.00 1.64 (1.37 – 1.96) p<0.0001	7756 (64.83%) 4208 (35.17%)	1373 (17.70%) 962 (22.86%)	1.00 1.38 (1.26 – 1.51) P<0.0001
Weekly frequency of using public or shared transport	None Any	12,469 (72%) 4787 (28%)	326 (2.61%) 176 (3.68%)	1.00 1.42 (1.18 – 1.71) P=0.0003	5902 (52%) 5511 (48%)	219 (3.7%) 274 (4.9%)	1.00 1.36 (1.13 – 1.62) P=0.0009	6,868 (57.41%) 5,096 (42.59%)	1212 (17.65%) 1123 (22.04%)	1.00 1.32 (1.20 – 1.44) P<0.0001
Weekly frequency of any retail		7462 (43%) 9794 (57%) *>0–1 >1	203 (2.72%) 299 (3.05%)	1.00 1.13 (0.94 – 1.35) P=0.1967	482 (4%) 2022 (18%) 5745 (50%) 3164 (28%) *0 >0 – 1 >1 – 3 > 3	14 (2.9%) 110 (5.4%) 262 (4.6%) 107 (3.4%)	0.85 (0.49 – 1.50) 1.64 (1.25 – 2.16) 1.37 (1.09 – 1.72) 1.00 P=0.0010	6029 (50.39%) 5935 (49.61%) 0 - 2 >2	1219 (20.22%) 1116 (18.80%)	1.09 (0.99 – 1.19) 1.00 P=0.0508
Weekly frequency of other non- household activities	0—3 More than 3	9769 (57%) 7487 (43%)	266 (2.72%) 236 (3.15%)	1.00 1.16 (0.97 – 1.39) P=0.0973	-	-	-	-	-	-
Indoor hospitality	None Any	-	-	-	6531 (57%) 4882 (43%)	258 (3.9%) 235 (4.8%)	1.00 1.23 (1.03 – 1.47) P=0.0254	2,772 (23.17%) 9192 (76.83%)	398 (14.36%) 1937 (21.07%)	1.00 1.59 (1.42 – 1.79) P<0.0001
Outdoor hospitality	None Any	-	-	-	6777 (59%) 4636 (41%)	276 (4.1%) 217 (4.7%)	1.00 1.16 (0.96 – 1.39) P=0.1179	8383 (70.07%) 3581 (29.93%)	1578 (18.82%) 757 (21.14%)	1.00 1.16 (1.05 – 1.27) P=0.0036
Indoor leisure	None Any	-	-	-	6713 (59%) 4700 (41%)	258 (3.8%) 235 (5%)	1.00 1.32 (1.01 – 1.58) P=0.0029	7252 (60.62%) 4712 (39.38%)	1231 (16.97%) 1104 (23.43%)	1.00 1.49 (1.37 – 1.64) P<0.0001
Outdoor leisure	None Any	-	-	-	10,570 (93%) 843 (7%)	449 (4.3%) 44 (5.2%)	1.00 1.24 (0.90 – 1.71) p=0.1943	11,092 (92.71%) 872 (7.29%)	2138 (19.28%) 197 (22.59%)	1.00 1.22 (1.04 – 1.44) P=0.0193
Non-social activity	None Any	-	-	-	7469 (65%) 3944 (35%)	351 (4.7%) 142 (3.6%)	1.00 0.76 (0.62 – 0.92) P=0.0053	7832 (65.46%) 4132 (34.54%)	1515 (19.34%) 820 (19.85%)	1.00 1.03 (0.94 – 1.14) P=0.5110

**Table 3.** Activity infection odds, unadjusted and adjusted (sex, region, vaccine status, living: alone; with children; in deprived area).

		Restriction period				Period after restriction				Omicron			
Activity	Frequency	Number (%) in cohort (N=17,256)	Covid infection (%) N=502	Unadjusted OR (95% CI), p	Adjusted OR (95% CI), p	N=11,413 (% in category)	Number of infections n=493(% within category)	Unadjusted OR (95% CI), p	Adjusted OR, (95% CI), p (n=11,231)	N=11, 964 (% in category)	Number of infections n= 2,335 (% within category)	Unadjusted OR (95% CI), p	Adjusted OR, (95% CI), p
Essential shopping	0 - 1 >1 -2 >2	2,718 (16%) 10,456 (61%) 4,082 (24%) *0, >0-2, >2	70 (2.6%) 299 (2.9%) 133 (3.3%)	1.00 1.11 (0.86 – 1.45) 1.27 (0.95 – 1.45) P=0.2344	1.00 1.07 (0.82 – 1.39) 1.18 (0.88 – 1.59) P=0.5010	3,756 (33%) 3,877 (34%) 3,780 (33%)	182 (5%) 180 (5%) 131 (3%)	1.00 0.96 (0.77 – 1.18) 0.71 (0.56 – 0.87) P=0.0052	1.00 1.02 (0.82 – 1.26) 0.72 (0.57 – 0.92) P=0.0072	3,841 (32%) 4,022 (34%) 4,101 (34%)	758 (19.7%) 842 (20.9%) 735 (17.9%)	1.00 1.08 (0.96 – 1.20) 0.89 (0.79 – 0.99) P=0.0025	1.00 1.09 (0.98 – 1.22) 0.89 (0.79 – 0.99) P=0.0013
Non- essential shopping	None Up to once More than once	13,523 (78%) 3,733 (22%) *None Any	398 (2.9%) 104 (2.8%)	1.00 0.95 (0.76 – 1.18) P=0.6112	1.00 1.00 (0.80 – 1.25) P=0.9816	4,073 (36%) 5,224 (46%) 2,116 (19%)	183 (4%) 222 (4%) 88 (4%)	1.00 0.94 (0.77 – 1.15) 0.92 (0.71 – 1.19) P=0.7832	1.00 0.93 (0.76 – 1.14) 0.89 (0.68 – 1.16) P=0.6399	4,573 (38%) 5,405 (45%) 1,986 (17%)	861 (18.8%) 1,088 (20.1%) 386 (19.4%)	1.00 1.09 (0.98 – 1.20) 1.04 (0.91 – 1.19) P=0.2613	1.00 1.07 (0.97 – 1.18) 1.02 (0.89 – 1.17) P=0.4135
Indoor pub, bar, club	None Up to once More than once	17,045 (99%) 211 (1%) *indoor and outdoor combined during restriction period	497 (2.9%) 5 (2.4%)	1.00 0.81 (0.33 – 1.97) P=0.6282	1.00 0.82 (0.33 – 2.04) P=0.6672	6,137 (54%) 3,373 (29%) 1,903 (17%)	243 (4%) 147 (4%) 103 (5%)	1.00 1.11(0.89 – 1.36) 1.39 (1.09 – 1.76) P=0.0288	1.00 1.14 (0.92 – 1.40) 1.45 (1.13 – 1.84) P=0.0138	6,709 (56%) 3,513 (29%) 1,742 (15%)	1,149 (17.13%) 799 (22.74%) 387 (22.22%)	1.00 1.42 (1.29 – 1.58) 1.38 (1.21 – 1.57) P<0.001	1.00 1.43 (1.29 – 1.58) 1.43 (1.26 – 1.63) P<0.0001
Outdoor pub, bar, club	None At least once	*indoor and outdoor combined during restriction period	-	-	-	9,088 (80%) 2,325 (20%)	387 (4%) 106 (5%)	1.00 1.07 (0.86 – 1.34) P=0.5271	1.00 1.09 (0.87 – 1.36) P=0.4653	10,287(86%) 1,677 (14%)	1,972 (19.17%) 363 (21.65%)	1.00 1.16 (1.03 – 1.32) P=0.0190	1.00 1.19 (1.05 – 1.35) P=0.0089
Indoor restaurant, café, canteen	None Up to once More than once	16,648 (96%) 608 (4%) *indoor and outdoor combined during restriction period; None/Any categories	489 (2.9%) 13 (2.1%)	1.00 0.72 (0.41 – 1.26) P=0.2280	1.00 0.71 (0.40 – 1.26) P=0.2240	3,325 (29%) 5,320 (47%) 2,768 (24%)	119 (4%) 263(5%) 111 (4%)	1.00 1.40 (1.12 – 1.75) 1.13 (0.86 – 1.47) P=0.0063	1.00 1.38 (1.11 – 1.73) 1.14 (0.87 – 1.49) P=0.0120	3,840 (32%) 5,578 (47%) 2,546 (21%)	626 (16.30%) 1,128 (20.22%) 581 (22.82%)	1.00 1.30 (1.17 – 1.45) 1.52 (1.24 – 1.72) P<0.0001	1.00 1.29 (1.16 – 1.44) 1.52 (1.34 – 1.73) P<0.0001
Outdoor restaurant, café, canteen	None At least once in 3 months	*indoor and outdoor combined during restriction period	-	-	-	8,147 (71%) 3,266 (29%)	342 (4%) 151 (5%)	1.00 1.11(0.91 – 1.35) P=0.3153	1.00 1.14 (0.94 – 1.39) P=0.1939	9,416 (79%) 2,548 (21%)	1,806 (19.18%) 529 (20.76%)	1.00 1.10 (0.99 – 1.23) P=0.0756	1.00 1.11 (0.99 – 1.23) P=0.0776
Indoor party	None At least once in 3 months	17,205 (99%) 51 (<1%) *indoor and outdoor combined during restriction period	501 (2.9%) 1 (1.9%)	1.00 0.66 (0.09 – 4.84) P=0.6684	1.00 0.56 (0.08 – 4.07) P=0.5254	9,967 (87%) 1,446 (13%)	408 (4%) 85 (6%)	1.00 1.46 (1.15 – 1.86) P=0.0028	1.00 1.38 (1.08 – 1.77) P=0.0124	10,871 (91%) 1,093 (9%)	2,062 (18.97%) 273 (24.98%)	1.00 1.42 (1.23 – 1.64) P<0.0001	1.00 1.39 (1.19 – 1.61) P<0.0001
Outdoor party	None At least once in 3 months	*indoor and outdoor combined during restriction period	-	-	-	10,959 (96%) 454 (4%)	474 (4%) 19 (4%)	1.00 0.97 (0.60 – 1.54) P=0.8850	1.00 0.91 (0.55 – 1.49) P=0.7110	11,745 (98%) 219 (2%)	2,286 (19.46%) 49 (22.37%)	1.00 1.19 (0.87 – 1.64) P=0.2898	1.00 1.19 (0.86 – 1.66) P=0.3025
Gym/indoor sport	None At least once in 3 months	16,954 (98%) 302 (2%)	491 (2.9%) 11 (3.6%)	1.00 1.27 (0.69 – 2.33) P=0.4610	1.00 1.20 (0.65 – 2.22) P=0.5657	8,894 (78%) 2,519 (22%)	361 (4%) 132 (5%)	1.00 1.31(1.07 – 1.60) P=0.0118	1.00 1.32 (1.07 – 1.63) P=0.0105	9,145 (76%) 2,819 (24%)	1,634 (17.87%) 701 (24.87%)	1.00 1.52 (1.38 – 1.68) P<0.0001	1.00 1.48 (1.33 – 1.64) P<0.0001
Team sport outdoors	None At least once	17,089 (99%) 167 (1%)	496 (2.9%) 6 (3.59%)	1.00 1.25 (0.55 – 2.83) P=0.6100	1.00 1.29 (0.56 – 2.96) P=0.5583	10,570 (93%) 843 (7%)	449 (4%) 44 (5%)	1.00 1.24 (0.90 – 1.71) P=0.1943	1.00 1.23 (0.89 – 1.71) P=0.2269	11,092 (93%) 872 (7%)	2,138 (19.28%) 197 (22.59%)	1.00 1.22 (1.04 – 1.44) P<0.0193	1.00 1.22 (1.03 – 1.44) P=0.0240
Theatre, cinema, concert, sports event	None At least	17,175 (99%) 81 (<1%)	498 (2.9%) 4 (4.9%)	1.00 1.74 (0.63 – 4.77) P=0.3207	1.00 1.74 (0.63 – 4.84) P=0.3229	8,250 (72%) 3,163 (28%)	336 (4%) 157 (5%)	1.00 1.23 (1.01 – 1.49) P=0.0387	1.00 1.23 (1.01 – 1.49) P=0.0464	9,095 (76%) 2,869 (24%)	1,679 (18.46%) 656 (22.87%)	1.00 1.31 (1.18 – 1.45) P<0.0001	1.00 1.28 (1.15 – 1.42) P<0.00001
Hairdresser, barber, beautician	None At least once in 3 months	16,797 (97%) 459 (3%)	494 (2.9%) 8 (1.7%)	1.00 0.59 (0.29 – 1.18) P=0.1046	1.00 0.59 (0.29 – 1.19) P=0.1123	7,469 (65%) 3,944 (35%)	351 (5%) 142 (4%)	1.00 0.76 (0.62 – 0.92) P=0.0053	1.00 0.76 (0.62 – 0.93) P=0.0078	7,832 (65%) 4,132 (35%)	1,515 (19.34%) 820 (19.85%)	1.00 1.03 (0.94 – 1.14) P=0.5110	1.00 1.04 (0.95 – 1.15) P=0.3931
Went to an outdoor market **	None Any	16,163 (94%) 1,093 (6%)	470 (2.9%) 32 (2.9%)	1.00 1.01 (0.70 – 1.45) P=0.9699	1.00 0.98 (0.68 – 1.42) P=0.9221	*not measured	-	-	-	*not measured	-	-	-
Went to a place of worship	None Any	16,688 (97%) 568 (3%)	486 (2.9%) 16 (2.8%)	1.00 0.97 (0.58 – 1.60) P=0.8937	1.00 0.96 (0.58 – 1.59) P=0.8655	*not measured	-	-	-	*not measured	-	-	-
Visited friends or family **	None Up to once More than once	*not measured	-	-	-	*not measured	-	-	-	3,339 (28%) 5,368 (45%) 3,257 (27%)	605 (18.12%) 1,109 (20.66%) 621 (19.07%)	1.00 1.18 (1.05 – 1.31) 1.06 (0.94 – 1.21) P=0.0108	1.00 1.18 (1.06 – 1.33) 1.08 (0.95 – 1.23) P=0.0099
Attended a wedding or funeral **	None Any	*not measured	-	-	-	*not measured	-	-	-	10,441 (87%) 1,523 (13%)	2.015 (19.30%) 320 (21.01%)	1.00 1.11 (0.97 – 1.27) P=0.1182	1.00 1.13 (0.98 – 1.29) P=0.0854

**not included in composites variables

### Activities associated with infection over time

**Figure 1.**
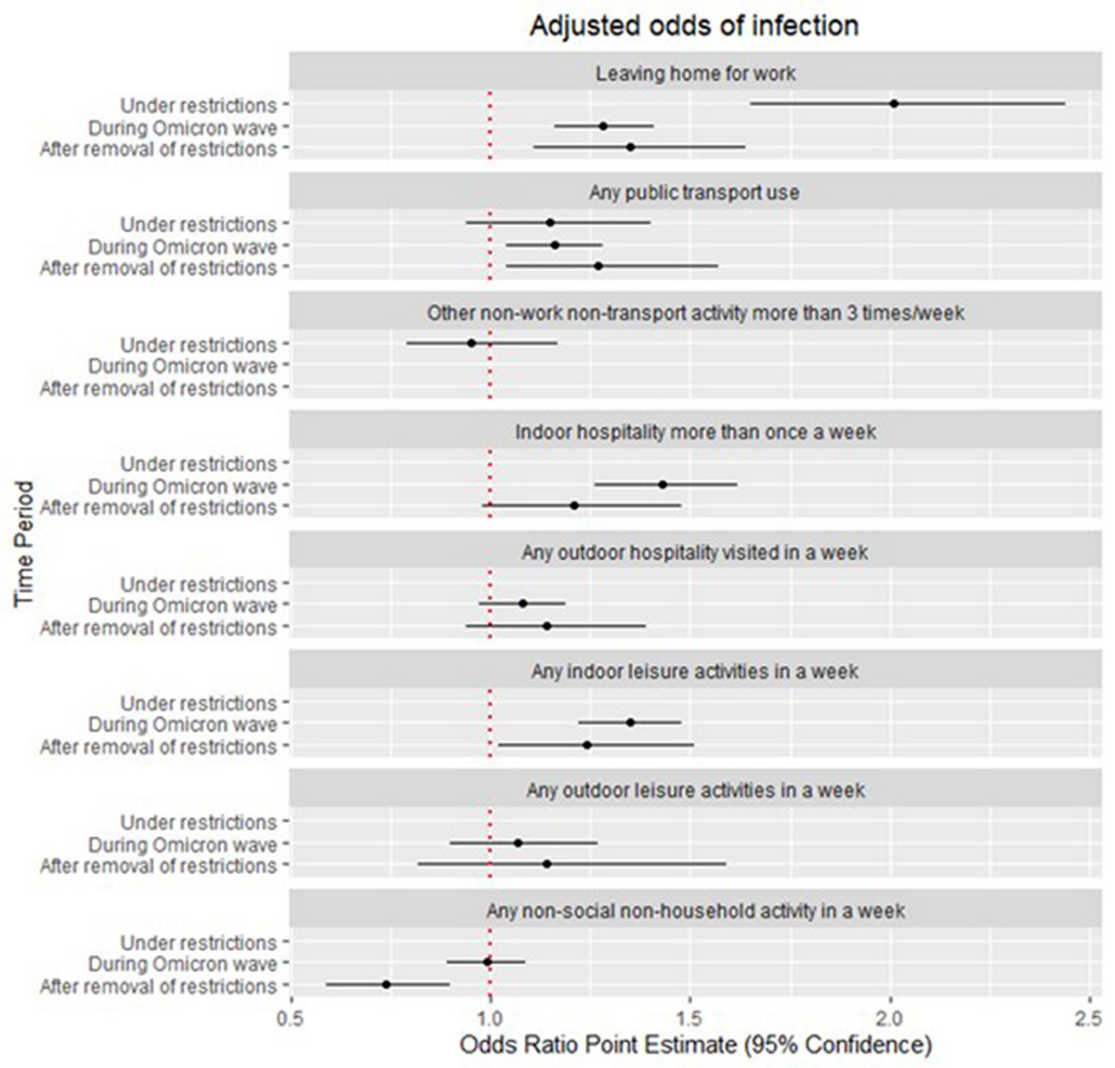
Adjusted odds of infection by activity throughout the waves. *Other non-work non-transport activities were disaggregated by Indoor and Outdoor activities for the periods After the removal of restrictions and During the Omicron wave.

During the period under intense restrictions, after multivariate adjustments, there was strong evidence that leaving home to go to work or education [adjusted odds ratio (aOR) 2.01 (1.65–2.44)] carried the greatest infection risk, with some evidence that using public transport (aOR 1.15 (0.94–1.40) also carried a risk ( Table 4, Figure 1). During this period when indoor social activities were largely curtailed, hospitality and leisure were not important risk factors for infection. During the period immediately following the lifting of public health restrictions, the risk associated with leaving home to go to work or education (aOR 1.35 (1.11–1.64) reduced in magnitude but remained the activity with the greatest infection risk while the risk associated with using public transport increased in magnitude and significance (aOR 1.27 (1.04–1.57). During this period after the removal of restrictions, indoor hospitality (aOR 1.21 (0.98–1.48)) and indoor leisure venues (aOR 1.24 (1.02–1.51)) became increasingly important risk factors. During the Omicron wave, leaving home for work (aOR 1.28 (1.16–1.41) and public transport (aOR 1.16 (1.04–1.28) were associated with a slightly lower, but significant infection risk than in the previous period while the risk of infection among participants using indoor hospitality (aOR 1.43 (1.26–1.62) and leisure venues (aOR 1.35 (1.22–1.48) increased.

**Table 4.** Infection odds and PAFs (adjustments: sex, region, vaccine status, living: alone; with children; in deprived area).

		Restriction period		Post lifting of national restrictions		Omicron wave	
Activity	Weekly frequency	Adjusted odds ratio (95% CI), p	Population attributable fractions (weighted) (95% CI)	Adjusted odds ratio (95% CI), p, (n=11,232)	Population attributable fractions (weighted) (95% CI)	Adjusted odds ratio (95% CI), p, (n=11,784)	Population attributable fractions (weighted) (95% CI)
Leaving home for work or education	No Yes	1.00 2.01 (1.65 – 2.44) P<0.0001	30.08 (21.-77 – 37.50)	1.00 1.35 (1.11 – 1.64) P=0.0029	16.51 (5.56 – 26.18)	1.00 1.28 (1.16 – 1.41) p<0.0001	10.92 (6.58 – 15.05)
Weekly frequency of using public or shared transport	None Any	1.00 1.15 (0.94 – 1.40) P=0.1814	4.77 (-2.50 – 11.52)	1.00 1.27 (1.04 – 1.57) P=0.0211	12.17 (1.59 – 21.61)	1.00 1.16 (1.04 – 1.28) P=0.0050	5.60 (1.65 – 9.40)
Weekly frequency of any retail	*	1.00 1.02 (0.84 – 1.24) P=0.8239	1.17 (-9.71 – 10.98)	1.05 (0.58 – 1.88) 1.83 (1.36 – 2.45) 1.51 (1.19 – 1.92) 1.00 P=0.0001	*not estimated	1.25 (1.13 – 1.37) 1.00 P<0.0001	*not estimated
Weekly frequency of other non-household activities	0—3 More than 3	1.00 0.95 (0.79 – 1.17) P=0.6776	*not plotted -1.96 (-11.64 – 6.89)	-	-		-
Indoor hospitality	None Any	-	-	1.00 1.21 (0.98 – 1.48) P=0.0710	7.26 (-0.90 – 14.77)	1.00 1.43 (1.26 – 1.62) P<0.0001	20.30 (13.14 – 26.37)
Outdoor hospitality	None Any	-	-	1.00 1.14 (0.94 – 1.39) P=0.1860	4.67 (-2.53 – 11.36)	1.00 1.08 (0.97 – 1.19) P=0.1578	1.68 (-0.69 – 3.99)
Indoor leisure	None Any	-	-	1.00 1.24 (1.02 – 1.51) P=0.0284	9.55 (0.71 – 17.60)	1.00 1.35 (1.22 – 1.48) P<0.0001	10.36 (6.91 – 13.68)
Outdoor leisure	None Any	-	-	1.00 1.14 (0.82 – 1.59) P=0.4521	1.14 (-1.96 – 4.16)	1.00 1.07 (0.90 – 1.27) P=0.4391	0.47 (-0.73 – 1.65)
Non-social activity	None Any	-	-	1.00 0.74 (0.59 – 0.90) P=0.0031	-8.69 (-14.42 - -3.25) *not plotted	1.00 0.99 (0.89 – 1.09) P=0.7919	-0.34 (-2.87 – 2.13) not plotted

### Relative contribution of activities to overall infection over time

**Figure 2.**
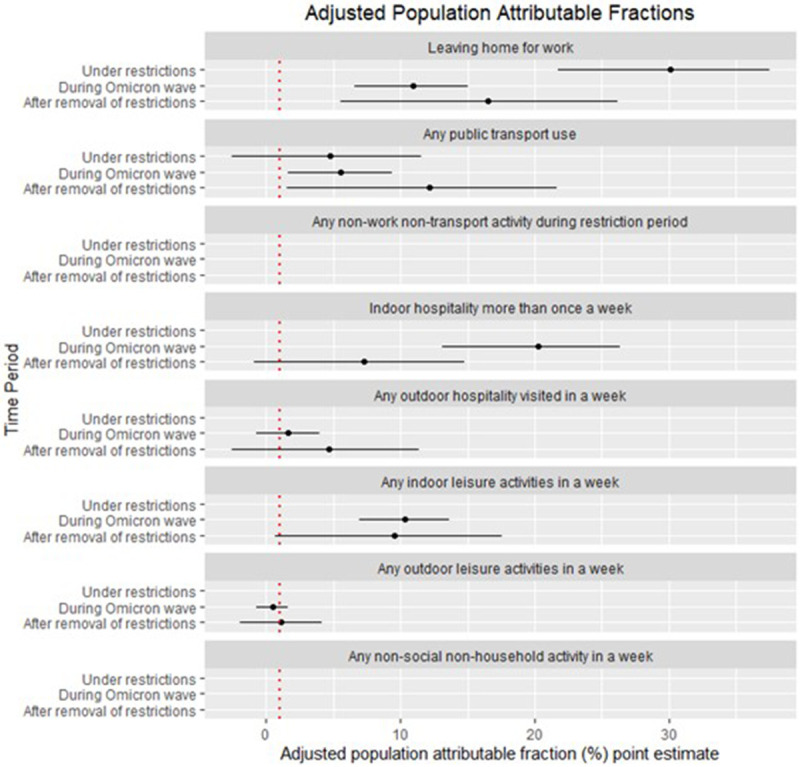
Adjusted Population Attributable Fractions (PAFs) by activity across the waves. **AFs below 0 are not plotted. Other non-work non-transport activities were disaggregated by Indoor and Outdoor activities for the periods After the removal of restrictions and During the Omicron wave.

During the period under intense restrictions, leaving home for work or education [population attributable fraction (PAF) 30% (21.77–37.50)] was the greatest contributor to infections, with public transport use (PAF 5% (-3–12%) contributing somewhat ( Table 4, Figure 2). Shopping contributed minimally during this period (PAF 1% (-9 to 11%) and other hospitality and leisure activities were not important. During the period immediately following the lifting of public health restrictions, leaving home for work (PAF 17% (6–26%) reduced in relative importance but continued to contribute significantly to infections. The relative contribution to infections of public transport use (PAF 12% (2–22%) became increasingly important as did the role of indoor hospitality (PAF 7% (-1–15%) and indoor leisure activities (PAF 10% (1–18%). During the Omicron wave, the relative role of leaving home for work (PAF (11% (7–15%) stayed the same as during the period immediately following the lifting of restrictions, but the role of public transport use (PAF 6% (2–9%) halved, through remained a significant contributor to overall infections. During this period of mostly no restrictions, the greatest contributor to infections was indoor hospitality (PAF 20% (13–26%), contributing nearly double the amount to infections than that contributed through leaving home for work. Indoor leisure use continued to contribute significantly (PAF 10% (7–14%), surpassing the role of public transport use during this period. 

Given the (unexpected) inverse relationship between shopping frequency and infection ( Table 3 and Table 4) during the period after the lifting of restrictions and during the Omicron wave, we did not calculate PAF for shopping. 

When additionally controlling for age ( Table 5), the relative contribution to infections of work was reduced under all three levels of national restrictions [in the period under restrictions (PAF 16% (17–34%), most notable during the period following the lifting of restrictions (PAF 3% (-10–15%)) and during the Omicron wave (PAF 6% (1–11%)]. 

**Table 5.** Infection odds and PAFs (adjustments: age, sex, region, vaccine status, living: alone; with children; in deprived area).

		Restriction period		Post lifting of national restrictions		Omicron wave	
Activity	Weekly frequency	Adjusted odds ratio additionally adjusted for age (95% CI), p, (n=16, 931)	Population attributable fractions when adjusting for age (weighted) (95% CI)	Adjusted odds ratio additionally adjusted for age (95% CI), p	Population attributable fractions when adjusting for age (weighted) (95% CI)	Adjusted odds ratio additionally adjusted for age (95% CI), p, (n=11,784)	Population attributable fractions when adjusting for age (weighted) (95% CI)
Leaving home for work or education	No Yes	1.00 1.79 (1.45 – 2.21) P<0.0001	25.80 (16.64 – 33.95)	1.00 1.06 (0.85-1.30) P=0.6181	3.25 (-10.25 – 15.10)	1.00 1.15 (1.03 – 1.28) P=0.0119	6.17 (1.31 – 10.80)
Weekly frequency of using public or shared transport	None Any	1.00 1.15 (0.94 – 1.41) P=0.1699	4.88 (-2.36 – 11.60)	1.00 1.27 (1.03 – 1.56) P=0.0260	11.85 (1.18 – 21.37)	1.00 1.15 (1.04 – 1.27) P-0.0084	5.13 (1.27 – 8.85)
Weekly frequency of any retail		1.00 1.03 (0.85 – 1.25) P=0.7618	1.59 (-9.24 – 11.35)	0.99 (0.55 – 1.79) 1.79 (1.33 – 2.41) 1.49 (1.18 – 1.89) 1.00 P=0.0002	*not estimated	1.25 (1.13 – 1.37) 1.00 P<0.0001	* not estimated
Weekly frequency of other non-household activities	0—3 More than 3	1.00 0.95 (0.78 – 1.16) P=0.6375	not estimated due to lack of significance		-	-	-
Indoor hospitality	None Any	-	-	1.00 1.22 (0.99 – 1.49) P=0.0567	7.64 (-0.49 – 15.12)	1.00 1.44 (1.27 – 1.63) P<0.0001	19.59 (12.87 – 25.79%)
Outdoor hospitality	None Any	-	-	1.00 1.11 (0.91 – 1.35) P=0.3091	3.62 (-3.63 – 10.36)	1.00 1.07 (0.97 – 1.19) P=0.1876	1.50 (-0.77% - 3.72)
Indoor leisure	None Any	-	-	1.00 1.21 (0.99 – 1.47) P=0.0621	8.24 (-0.73 – 16.40)	1.00 1.33 (1.21 – 1.47) P<0.0001	9.66 (6.30 – 12.90)
Outdoor leisure	None Any	-	-	1.00 1.14 (0.82 – 1.59) P=0.4532	1.17 (-2.01 – 4.25)	1.00 1.07 (0.89 – 1.26) P<0.4861	0.41 (-0.75 – 1.54)
Non-social activity	None Any	-	-	1.00 0.74 (0.60 – 0.91) P=0.0042	-8.37 (-14.08 - -2.94) *not estimated	1.00 0.98 (0.89 – 1.08) P=0.9950	-0.01% (-2.42 – 2.35) *not estimated

When additionally adjusting for age ( Table 5), the only activity to be affected with much importance was leaving home for work, whose infection odds and relative contributory role to overall infections was reduced across all periods. During the period under restrictions the odds of infection associated with leaving home for work or education reduced slightly (OR 1.79 [1.45 – 2.21]), but work remained the greatest contributor (PAF 26% [17 – 34%] to infections during this period. The adjustment for age had the greatest impact on the role of work during the period immediately following the lifting of restrictions, with the odds of infection associated with leaving home for work becoming non-significant ((OR 1.06 [0.85 – 1.30] and its contributory role reduced to 3% PAF (-10 to 15%). During the Omicron wave, the additional adjustment for age halved the odds associated with leaving home for work (OR 1.15 (1.03 – 1.28) and its contributory role to infections (PAF 6% (1 – 11%). For all other activities, the infection risk and the contributory role to overall infections were little affected when additionally adjusting for age. 

### Individual activities associated with infection over time

Throughout the pandemic, the magnitude of infection odds associated with individual transport methods ( Table 6) increased as a greater proportion of participants used public transport. Notably, bus use carried an increased infection odds of between OR 1.22 (0.89–1.67) when 7% of participants used a bus in the restriction period and OR 1.27 (1.14–1.41) when one quarter of participants used a bus during the Omicron wave. Similarly, there was a consistent increased odds of infection with overground train or tram use throughout the pandemic, gaining in strength and significance over time, up to OR 1.45 (1.13–1.86) during the Omicron period. Infection odds associated with underground use oscillated from nearly double during the restriction phase (OR 1.87, 1.26–2.77) when only 3% of participants used an underground train, to OR 1.14 (0.87–1.51) in the period after the removal of restrictions, regaining strength and significance (OR 1.35, 1.16–1.56) during the Omicron wave. The risk associated with airplane use was high (OR 1.39, 1.20–1.62). 

**Table 6.** Transport infection odds, unadjusted and adjusted (sex, region, vaccine status, living: alone; with children; in deprived area).

		Period under restrictions				Post lifting of restrictions				Omicron wave			
Characteristic	Frequency	Number in cohort (%) (N=17,256)	Number of infections (N=502) (% within category)	Unadjusted OR, 95% CI, p	Adjusted OR, 95% CI, p (n=16,931)	N=11,413(% in category)	Number of infections n= 493 (% within category)	Unadjusted OR	Adjusted OR, 95% CI, p (n=11,231)	N=11, 964 (% in category)	Number of infections n= 2,335 (% within category)	Unadjusted OR	Adjusted OR, 95% CI, p
Shared car	None Any	13,705 (79%) 3,551 (21%) *shared car and taxi combined	375 (2.7%) 127 (3.6%)	1.00 1.32 (1.07 – 1.62) P=0.0096	1.00 1.26 (1.02 – 1.56) P=0.0314	4,634 (41%) 6,779 (59%)	211 (5%) 282 (4%)	1.00 0.91 (0.76 – 1.09) P=0.3112	1.00 0.99 (0.83 – 1.20) P=0.9642	5,040 (42%) 4,862 (41%) 2,062 (17%)	890 (17.7%) 1,004 (20.7%) 441 (21.4%)	1.00 1.21 (1.09 – 1.34) 1.27 (1.12 – 1.44) P=0.0001	1.00 1.22 (1.11 – 1.36) 1.34 (1.18– 1.53) P<0.0001
Taxi	None Any	*combined with shared car in restriction period	-	-	-	9,217 (81%) 2,196 (19%)	376 (4%) 117 (5%)	1.00 1.32 (1.07 – 1.64) P=0.0117	1.00 1.28 (1.03 – 1.59) P=0.0321	10,128 (85%) 1,836 (15%)	1,922 (18.9%) 413 (22.5%)	1.00 1.24 (1.09 – 1.39) P=0.0006	1.00 1.22 (1.08 – 1.38) P=0.0014
Bus	None Any	16,024 (93%) 1,232 (7%)	448 (2.8%) 54 (4.4%)	1.00 1.59 (1.19 – 2.13) P=0.0028	1.00 1.22 (0.89 – 1.67) P=0.2098	8,250 (72%) 3,163 (28%) * None Any	342 (4%) 151 (5%)	1.00 1.16 (0.95 – 1.41) P=0.1432	1.00 1.27 (1.03 – 1.56) P=0.0291	9,007 (75%) 2,957 (25%)	1,675 (18.6%) 660 (22.3%)	1.00 1.26 (1.14 – 1.39) P<0.001	1.00 1.27 (1.14 – 1.41) P<0.0001
Overground train	None Any	16,629 (96%) 627 (4%) *combined train or tram in restriction period	473 (2.8%) 29 (4.6%)	1.00 1.66 (1.13 – 2.43) P=0.0159	1.00 1.25 (0.83 – 1.86) P=0.2968	8,694 (76%) 2,719 (24%) *overground train and tram combined	344 (4%) 149 (5%)	1.00 1.41 (1.16 – 1.71) P=0.0009	1.00 1.41 (1.14 – 1.74) P=0.0017	9,737 (81%) 2,227 (19%)	1,824 (18.7%) 511 (22.9%)	1.00 1.29 (1.16 – 1.44) P<0.0001	1.00 1.27 (1.13 – 1.43) P=0.0001
Overround tram	None Any	*combined train or tram in restriction period	-	-	-	*combined with overground train	-		-	11,612 (97%) 352 (3%)	2,243 (19%) 92 (26.1%)	1.00 1.48 (1.16 – 1.88) P=0.0022	1.00 1.45 (1.13 – 1.86) P=0.0044
Underground train	None Any	16,735 (97%) 521 (3%)	466 (2.8) 36 (6.9%)	1.00 2.59 (1.82 – 3.68) P<0.0001	1.00 1.87 (1.26 – 2.77) P=0.0032	9,709 (85%) 1,704 (15%)	409 (4%) 84 (5%)	1.00 1.18 (0.93 – 1.49) P=0.1870	1.00 1.14 (0.87 – 1.51) P=0.3406	10,512 (88%) 1,452 (12%)	1,984 (18.9%) 351 (24.2%)	1.00 1.37 (1.20 – 1.56) P<0.0001	1.00 1.35 (1.16 – 1.56) P=0.0001
Airplane	None Any	17,114 (99%) 142 (1%)	497 (2.9%) 5 (3.5%)	1.00 1.22 (0.49 – 2.99) P=0.6729	1.00 1.14 (0.46 – 2.79) P=0.7864	10,338 (91%) 1,075 (9%)	443 (4%) 50 (5%)	1.00 1.09 (0.81 – 1.47) P=0.5783	1.00 1.04 (0.76 – 1.41) P=0.8259	10,924 (91%) 1,040 (9%)	2,073 (18.9%) 262 (25.2%)	1.00 1.44 (1.24 – 1.67) P<0.001	1.00 1.39 (1.20 – 1.62) P<0.0001

During the period under restrictions, individual activities ( Table 3) were undertaken by around 1% of participants and no individual activity was associated with a significant increased infection odds. When venues were no longer under public health restrictions, visiting an indoor bar, club or pub carried a consistently higher odds of infection [OR 1.45 (1.13–1.84) after the lifting of restrictions and OR 1.43 (1.26–1.63) during the Omicron wave] than visiting its indoor equivalent [OR 1.09 (0.87–1.36) after the lifting of restrictions and OR 1.19 (1.05–1.35) during the Omicron wave]. A similar magnitude of elevated risk was associated with indoor restaurant use [OR 1.38 (1.11–1.73) after the lifting of restrictions and OR 1.52 (1.34–1.73) during the Omicron wave] but not with its outdoor equivalent [OR 1.14 (0.94–1.39) immediately after the lifting of restrictions and OR 1.11 (0.99–1.23) during the Omicron wave], and with attending an indoor party [OR 1.38 (1.08–1.77) after the lifting of restrictions and OR 1.39 (1.19 – 1.61) during the Omicron wave] but not with attending a party outdoors [OR 0.91 (0.55–1.49) after the lifting of restrictions and OR 1.19 (0.86–1.66), during the Omicron wave). Indoor sport carried a significantly higher risk than outdoor sport during both periods and visiting a theatre or concert increased risk by around a quarter (OR 1.23 (1.01–1.49) after the lifting of restrictions and OR 1.28 (1.15–1.42) during the Omicron wave].

## Discussion

We found that during periods of intense restrictions (October 2020–May 2021), work and public transport were important risk factors for SARS-CoV-2 acquisition and contributors to overall infections, but hospitality and leisure were not important. During the period when most public health restrictions were lifted (September–mid-December 2021) work, public transport, indoor hospitality and indoor leisure venues became important contributors to transmission. During the Omicron wave (December 2021–April 2022), characterised by no restrictions for the majority of the period and a return to activity levels closer to those of pre-pandemic, work and public transport were still significant risk factors but diminished in importance with indoor hospitality and leisure venues increasing in importance. 

The sizeable contributory role to infections of leaving home for work during the period under restrictions relative to other activities demonstrates the effectiveness of the restriction measures. One of the few reasons individuals in the UK were allowed to leave home during the period under restrictions was for work that could not feasibly be done from home such as being an essential worker. Such roles included working in healthcare, as a carer or transport worker. These roles were demonstrated early in the pandemic to carry considerable increased transmission risk and our findings that the greatest contribution to infections during the restriction period was among individuals leaving home for work fits with this. We have previously found that front line occupations were most at risk during the early stages of the pandemic, but occupation became a less important driver of infection risk in later waves of the pandemic ^20^. The lack of contribution to infections of hospitality and leisure venues during the restriction period compared to their important contribution after the removal of restrictions highlights the effectiveness of the closure of these venues when the country was faced with a respiratory virus of high pathogenicity. As increasing numbers of participants returned to visiting indoor venues during the period immediately after the removal of restrictions and some months later during the Omicron wave, the risk of infection increased. The larger contribution to infections of indoor hospitality during the Omicron period compared to the role of work during that period can be partly explained by the near double proportion of participants attending indoor hospitality venues (77%) *versus* the proportion leaving home for work (35%) and may also relate to the increased transmissibility of Omicron. As restrictions were lifted, how people became infected was largely based on the extent to which people used the new freedoms to use leisure and hospitality venues.

Indoor hospitality and leisure activities significantly increased risk of infection but this risk was diminished and not statistically significant for outdoor hospitality and leisure activities, (likely due to massively higher ventilation in outdoor settings). This suggests that measures to allow outdoor use of such venues may be a proportionate approach to balancing risk of infection whilst avoiding total closure of such venues. However, a relatively small proportion of participants visited outdoor hospitality or leisure venues compared to the proportion undertaking indoor hospitality and leisure activities limiting the power to accurately measure risk in outdoor settings. 

We hypothesised that age is a very strong determinant of social interactions and particularly for whether or not people work, and that therefore controlling for age would reduce or remove many of the associations found. However, with the exception of leaving home for work, the effect of controlling for age was minimal. The notable reduction in risk related to leaving home for work when additionally adjusting for age suggests that an important part of the reduced risk of infection in older adults relates to reduced exposure to work in those over retirement age. 

Odds of infection were paradoxically found to decrease with more frequent shopping, except in the period under restrictions. We hypothesize that shopping once a week may be associated with a longer ‘weekly shop’ in larger venues, consequently presenting greater risk than more frequent, shorter shops in smaller stores with a lower capacity.

### Other work

Findings in this study update a previous investigation during the restriction period to include more identified cases and further adjustment for potential confounding; essential activities such as attending work and using transport remained important contributors to risk ^17^. Findings broadly corroborate and extend results identified in other studies investigating the relationship between activities and SARS-CoV-2 infection but extend this by using consistent methodology through multiple periods of the pandemic, with comprehensive measurement of the range of settings where exposure can occur and adjustment for important confounders.

Findings regarding the period during which most restrictions were lifted are similar to other studies investigating periods with relatively relaxed restrictions. During periods following the relaxation of restrictions, public transport was identified as a risk factor in studies from previous pandemic phases in the USA and France, although non-pharmaceutical interventions were more stringent in these settings than in England and Wales during the period in this study ^11, 12^. Similar to our own results, indoor hospitality and leisure venues were also identified to contribute to infection risk during periods of relaxed restrictions in France and Denmark ^12, 13, 15^. The persistent, though attenuated, relationship between attending work and transmission identified in the present study but not in previous literature may reflect differences in activity measurement, adjustment, and/or features of the pandemic including infection control within workplaces and prevalence of home working in different countries. Broadly, findings corroborate previous studies indicating that essential activities are the primary contributor to transmission during periods of stringent restrictions and that leisure and hospitality activities become increasingly important under periods of relaxed restrictions.

### Strengths and limitations

Self-reported activity surveys may be affected by recall and social desirability bias, however previous studies have supported the validity of self-reported contact survey data to reflect infection dynamics ^21^. To reduce participant burden and increase retention in the survey, we elected to use monthly surveys with detailed reporting of activities over a one week period prior to the survey, with much less detailed reporting in weekly surveys. This allowed comprehensive recording of a wide range of activities conducted in different settings and by averaging results from monthly surveys provided a measure of exposure over that period. The surveys asked about activities in the week before the survey to minimise recall bias. Measures of exposure behaviour in the incubation period prior to infection could theoretically be obtained from weekly behavioural surveys, and may facilitate stronger causal interpretation, but was complicated by isolation restrictions whereby contacts of known cases reduced exposure to non-household activities in the period before infection. Also, as stated above, the weekly surveys had considerably less detail. A strength of the study is that we sought to obtain good ascertainment of SARS-CoV-2 infections through both self-reported and linked data on test results from the national testing system, which allowed ascertainment of infections. However, these will depend on testing behaviours which may vary across groups potentially introducing biases.

Results may be affected by residual confounding - given challenges with adjustment for multiple complex sociodemographic and behavioural factors – and recall bias given that surveys required retrospective recall. These biases may provide an alternate explanation for the protective effect of personal care activities. Surveys were also limited in detail to reduce participant burden, and consequently could not capture detail of protective behaviours (e.g., face coverings and hand hygiene) or risk-relevant environmental features (e.g., ventilation) during public activities. 

Our comparison across the waves is subject to survivorship bias. Participants who died or suffered severe illness due to COVID-19 early in the pandemic could not or would be unlikely to contribute to activity analyses during the latter waves. However, as the overall proportion of these severe cases is low, this is unlikely to have had an important impact on findings. Our study may have been subject to selection bias whereby individuals who elect to be part of a research study examining associations between their activities and infection may have had different frequency of exposure to activities or differing self-protection behaviours such as mask wearing or hand washing than the general population. We partially addressed this through weighting PAFs to the national age structure of the population (accounting for the the older age structure of the Virus Watch population) but could not address other systematic differences in exposures in the cohort compared to the general population. For example, if Virus Watch contained a lower proportion of public transport users than the general public, then the proportion of infections attributed to public transport use within the cohort would likely be an underestimate of the proportion attributable to this exposure across the wider English population. 

## Conclusion and recommendations

We found that essential activities are the primary contributor to transmission during periods of stringent restrictions and that leisure and hospitality activities become increasingly important under periods of relaxed restrictions. The change in risk factors across the three periods of the pandemic through differing levels of national restrictions and resulting behaviour in England and Wales highlights the value of encouraging people to work from home, reduce public transport use and restrict visits to leisure and hospitality settings when the country was faced with a highly infectious virulent respiratory infection. Outdoor use of leisure and hospitality venues appeared to be safer than indoor use.

As population immunity has increased and the severity of COVID-19 has decreased, most countries have now moved to a phase of “living with Covid” with little or no restrictions. Improving ventilation in workplaces, hospitality and leisure venues may provide ongoing protection against transmission of COVID-19 and a range of other respiratory infections with minimal societal disruption. 

In the event of the emergence or re-emergence of a highly transmissible respiratory infection with appreciable mortality, these findings support the value of advice/restrictions to work from home where possible, workplace mitigations for those who cannot work from home and advice/restrictions to avoid indoor usage of hospitality and leisure venues as effective approaches to reduce transmission and associated mortality.

## Data Availability

As the Virus Watch dataset contains sensitive health data as well as other sensitive personal information, the raw data cannot be published publicly at the individual level. Individual record-level (excluding any data or variables originating from linkage via NHS Digital) are available on the Office for National Statistics Secure Research Service (SRS) [ https://ons.metadata.works/]. The data are available under restricted access and can be obtained by submitting a request directly to the SRS by searching for ‘Virus Watch’ and following the subsequent instructions.
