# Supplementary material for: The changing contributory role to infections of work, public transport, shopping, hospitality and leisure activities throughout the SARS-CoV-2 pandemic in England and Wales
Source: NIHR Open Res. 2023 Nov 3;3:58. [Version 1] doi: 10.3310/nihropenres.13443.1 (PMC11403290; doi:10.3310/nihropenres.13443.1)
Supplement: Peer review history

# Reviewer report: Reviewer response for version 1

**DOI:** 10.3310/nihropenres.14581.r30945

**Published:** 2024-09-15

**Authors:** Benjamin Wachtler

**Affiliations:** 1 Department of Epidemiology and Health Monitoring, Robert Koch Institute, Berlin, Germany

**Overall:**

 Thank you for giving me the opportunity to read and comment on this interesting research paper. The present study used data from the Virus Watch Community Cohort Study in England and Wales and aimed to investigate how work, public transport, shopping and hospitality and leisure activities contributed to SARS-CoV-2 infection risk during different periods with different public health restrictions. It thereby aims to assess the effectiveness of different public health interventions across different periods of the pandemic. Odds Ratios (OR) from multivariate logistic regression models and adjusted populations attributable fractions (aPAF) were estimated. Overall, the manuscript is well written and uses appropriate methods. I appreciate that the study aims to investigate the time varying effects of different activities by using high quality data and think that the research question is interesting and the results are still timely and needed for further debates around pandemic preparedness. However, I have some questions and comments that I would ask the authors to address to further improve their study and to reduce ambiguities.

1. It remains unclear what epidemiological design was used here. The authors used data from the Virus Watch Study for three different time periods. It remains unclear if the individuals may occur in several of these periods or only in one time period. Please indicate how many of the participant of each time period were the same or if these were different individuals. A explaining figure might help here. Please give at least short information about the sampling strategy, drop-outs and systematic non-response and how this might have affected the results and how these issues were handled.
2. If these three samples were independent samples, they should be described separately like it is done in table 1. Are there individual level information about socioeconomic position (SEP) available? Further information about the sociodemographic composition of the sample are desirable as we know, that SEP is a strong and time dependent predictor of SARS-CoV-2 infection. This is not mentioned in the manuscript but should be at least discussed. In addition, we know that the infection risk of different occupations changed between different periods of the pandemic, e.g. health care worker had a very high infection risk at the beginning of the pandemic that later was reduced by the introduction of sufficient personal protection equipment and vaccinations and during later phases of the pandemic e.g. construction worker had a higher infection risk. Please indicate how changing distributions of occupations between the samples might have infected your results.
3. To calculate PAFs is an interesting approach here but the underlying strong assumption of causality should be further discussed in this context. I have some doubts that causality can be assumed here. Please describe in more detail the approach that was used to calculate PAF.
4. I was wondering why you decided to calculate OR instead of using all available information of your data and calculate incidence rate ratios? Wouldn’t this be the ‘gold standard’ for assessing relative risks? Please explain your rationale and the potential limitations of this approach in more detail.
5. Please provide the reader with the DAG that was used to identify the adjustment set of the analyses (e.g. as supplementary material). The role of age in your analyses still remains unclear and I believe you should stratify the analyses by age to further explore the role of age in your study. In this context, please give the reader more background about the role of participants’ SEP. What was the rationale to include area level deprivation as a confounding variable? You are arguing that age is reflected by its affects on social behaviour. The same holds true for the SEP. We know that compliance with public health interventions is associated with SEP as well as infection risk and the possibility to reduce work-related and non-work-related physical contacts. I am missing a discussion of these questions in this paper.
6. Table 3 and table 4: I was wondering how the estimates given here were derived? Are all these estimates from one model? How do you rule out table II fallacy here? (Westreich D, Greenland S, 2013 [Ref-1])

**In detail:**

**Title: **The title appears to be very long. Is there the possibility to formulate a more concise title?

**Fig 1 and Fig. 2 **Both figures should include information about the adjustment sets used for the calculation in order to be interpretable without referring to the main text

**Discussion:**

 The discussion about the reduced importance of occupation as driver of infection risk in later waves of the pandemic should be discussed in more detail here. What may have driven this reduction in importance and what does this mean for public health interventions and pandemic preparedness?

Is the work clearly and accurately presented and does it cite the current literature?

Yes

If applicable, is the statistical analysis and its interpretation appropriate?

Partly

Are all the source data underlying the results available to ensure full reproducibility?

Yes

Is the study design appropriate and is the work technically sound?

Partly

Are the conclusions drawn adequately supported by the results?

Yes

Are sufficient details of methods and analysis provided to allow replication by others?

No

Reviewer Expertise:

Social Epidemiology, Health Inequalities in COVID-19, Public Health

I confirm that I have read this submission and believe that I have an appropriate level of expertise to confirm that it is of an acceptable scientific standard, however I have significant reservations, as outlined above.

## References

[1] : The table 2 fallacy: presenting and interpreting confounder and modifier coefficients. *Am J Epidemiol* .2013;177(4) : 10.1093/aje/kws412 292-8 10.1093/aje/kws412 23371353 PMC3626058

# Reviewer report: Reviewer response for version 1

**DOI:** 10.3310/nihropenres.14581.r31022

**Published:** 2024-09-15

**Authors:** Larese Filon Francesca

**Affiliations:** 1 Department of Medical Sciences, Universita degli Studi di Trieste, Trieste, Friuli-Venezia Giulia, Italy

The paper analyses factors associated to COVID-19 in a cohort of population during 2020-2021-2022. The paper is valuable; methods and statistical analysis are strong and well done. It deserve indexing. I suggest some improvements-

1. Please specify in the tables the 3 periods considered to increase readability
2. M&M please explain better the periods used for analysis: it is not clear to the reader
3. Effect of vaccination: would be interesting to discuss why the paper fails to find a protective effect for vaccination in the second period considered
4. I suggest to specify the number of doses of vaccine done in table 1 and to specify how many doses were considered as “vaccinates status yes” in the adjustment.
5. Did you consider the time of vaccination in relation to infections? I suggest to specify that in M&M section

Is the work clearly and accurately presented and does it cite the current literature?

Partly

If applicable, is the statistical analysis and its interpretation appropriate?

Yes

Are all the source data underlying the results available to ensure full reproducibility?

Partly

Is the study design appropriate and is the work technically sound?

Yes

Are the conclusions drawn adequately supported by the results?

Yes

Are sufficient details of methods and analysis provided to allow replication by others?

Partly

Reviewer Expertise:

Epidemiology of COVID-19 in workers

I confirm that I have read this submission and believe that I have an appropriate level of expertise to confirm that it is of an acceptable scientific standard, however I have significant reservations, as outlined above.

# Reviewer report: Reviewer response for version 1

**DOI:** 10.3310/nihropenres.14581.r30771

**Published:** 2024-01-08

**Authors:** Tsz Ho Kwan

**Affiliations:** 1 Jockey Club School of Public Health and Primary Care, The Chinese University of Hong Kong, Hong Kong, Hong Kong; 2 Stanley Ho Centre for Emerging Infectious Diseases, The Chinese University of Hong Kong, Hong Kong, Hong Kong

This study used the data collected from monthly household surveys in England and Wales to quantify the role of various activities in SARS-CoV-2 infection. The authors estimated and compared the activities' contributions to transmission across the three periods. The paper was well written, the methods were well constructed, and the discussion was supported by the results. This paper reflected the necessity of the stringent policies in place and the effects of lifting such restrictions. While the results were within expectations, it highlighted the importance of using household surveys to collect and evaluate the transmission risk of respiratory pathogens.

 There are some minor queries:

 - It was noted that the periods of data inclusion were of different lengths. Would this affect the results?

 - The periods of infection data were inconsistent as well. For the first period, there was a two-month period after the survey; the period became less than a month for the second period; and it was 0 day for the third period (30 March 2022). How would this affect the fair comparison and the results?

 - Would the authors confirm if the starting date of the infection data collection period 2 months earlier than the first period's survey period? It was noted that the recall period of the monthly survey was one month.

 - Interpreting insignificant results should be cautioned (page 13 on the PAFs, and page 14 on bus use).

 - It was noted age modified the importance of various types of activities, a stratified analysis may be useful to determine the effect across age groups. I might have missed the results but there was a discussion on this (page 17 second column second paragraph).

Is the work clearly and accurately presented and does it cite the current literature?

Yes

If applicable, is the statistical analysis and its interpretation appropriate?

Yes

Are all the source data underlying the results available to ensure full reproducibility?

Yes

Is the study design appropriate and is the work technically sound?

Yes

Are the conclusions drawn adequately supported by the results?

Yes

Are sufficient details of methods and analysis provided to allow replication by others?

Yes

Reviewer Expertise:

infectious diseases epidemiology

I confirm that I have read this submission and believe that I have an appropriate level of expertise to confirm that it is of an acceptable scientific standard.

# Reviewer report: Reviewer response for version 1

**DOI:** 10.3310/nihropenres.14581.r30943

**Published:** 2024-01-08

**Authors:** Mario Coccia

**Affiliations:** 1 IRCRES-CNR, Turin Research Area of the National Research Council, National Research Council of Italy, Torino, Italy

The changing contributory role to infections of work, public transport, shopping, hospitality and leisure activities throughout the SARS-CoV-2 pandemic in England and Wales

 The topics of this paper are  interesting, though well known. The structure and content must be revised, and results have to be  better explained by authors before to be reconsidered for publication.

 Title has to be shorter, but to indicate the period such as 2020-2021, etc. 

 Abstract has to clarify the goal and health and social policy implications to face next health emergencies. 

 Introduction has to better clarify the research questions of this study and provide more theoretical background. Authors have to better describe the different sources of transmission dynamics of COVID-19 (e.g., climate, air pollution, density, mobility, trade, etc.) and risk factors in society, which can  accelerate  diffusion of this novel coronavirus in environment, and after that they can focus on the topics of this study to provide a correct analysis for fruitful analysis and discussion (See suggested readings that must be all read and used in the text). 

 Methods of this study has to be better systematize.  The section of Materials and methods must be re-structured with following three sections to be clear:

 •    Sample and data

 •    Measures of variables. They have to be well clarified how they are normalized. 

 •    Models and Data analysis procedure. 

 Authors have to avoid a lot of subheadings that create fragmentation and confusion. If necessary, can use bullet points (same comments for section of results and all sections). 

 Results. 

 The paper has a lot of long and full  tables that are difficult to digest, some of them can be put in appendix and inserting in the text the most important ones to improve the readability…

 An aspect that is not clear is if these people infected are or not vaccinated. Only infections is a partial analysis, fatality rates also matters, for a complementary and robust analysis. 

 Results seem to be relevant but representation with figure 1 and 2 do not show the really effective differences under and after the removal of restrictions. Moreover, the level of infections alone is not sufficient, it should be associated with fatality rates considering an appropriate time lag from infection. We know that Omicron has a high rate of infections but mortality is lower than Delfa variants. Results have to be better clarified. 

 A main aspect to clarify is that transmission dynamics is associated with density of people, mainly in urban contexts. Wales and England have different density  and different cities of large size. It is not clear how data are normalized for comparative analyses and if they refer to urban or rural contexts. These differences should be clarified for better implications. 

 All tables and figures have to indicate the period under study. 

 Discussion. 

 First, authors have to synthesize the main results in a simple table to be clear for readers and then show what this study adds compared to other studies. Now reading the long description, reader loses and does not catch the difference of infections with and without restriction. 

 To  reiterate remove sub-headings that create a lot of fragmentation. Results have to be better explained also considering density of cities under study, level of pollution, duration of restrictions, etc. Now many factors of transmission dynamics are not discussed and reduce the value of this contribution for policy implications. 

 Conclusion has not to be a summary, but authors have to focus on manifold limitations of this study (moving here previous section) and provide suggestions of health, crisis management  and social policy, as well as how nations can prevent and manage next pandemics, with good governance,  with timely nonpharmaceutical measures of control and vaccination plans. 

 Overall, then, the paper is interesting, but many results well known. Theoretical framework is weak, and some results create confusion… structure of the paper has to be improved; study design, discussion and presentation of results have to be clarified using suggested comments.

 To be clear, I strongly suggest improving the paper,  by using all comments (suggested papers included to read and use all) that I will in-depth verify,  and maybe it can be considered.

 Suggested readings of relevant papers that have to be read and all inserted in the text and references.

 Benati I.,Coccia M. 2022. Effective Contact Tracing System Minimizes COVID-19 Related Infections and Deaths: Policy Lessons to Reduce the Impact of Future Pandemic Diseases. Journal of Public Administration and Governance, vol. 12, n. 3, pp. 19-33. DOI: https://doi.org/10.5296/jpag.v12i3.19834

 Ball P (2021) What the COVID-19 pandemic reveals about science, policy and society. Interface Focus 11:20210022. https:// doi. org/10. 1098/ rsfs. 2021. 0022

 Coccia M. 2023. Sources, diffusion and prediction in COVID-19 pandemic: lessons learned to face next health emergency[J]. AIMS Public Health, 2023, 10(1): 145-168. doi: 10.3934/publichealth.2023012

 Anttiroiko A-V (2021) Successful government responses to the pandemic: contextualizing national and urban responses to the COVID-19 outbreak in east and west. Int J E-Plan Res (IJEPR),IGI Global 10(2):1–17

 Coccia M. 2022. Preparedness of countries to face COVID-19 pandemic crisis: Strategic positioning and underlying structural factors to support strategies of prevention of pandemic threats, Environmental Research, Volume 203, n. 111678,  https://doi.org/10.1016/j.envres.2021.111678.

 Núñez-Delgado A., Bontempi E., Coccia M., Kumar M., Farkas K., Domingo, J. L. 2021. SARS-CoV-2 and other pathogenic microorganisms in the environment, Environmental Research, Volume 201, n. 111606, https://doi.org/10.1016/j.envres.2021.111606.

 Allen DW (2022) COVID-19 lockdown cost/benefits: a critical assessment of the literature. Int J Econ Bus 29(1):1–32. https:// doi. org/10. 1080/ 13571 516. 2021. 19760 51

 Coccia M. 2021. The impact of first and second wave of the COVID-19 pandemic: comparative analysis to support control measures to cope with negative effects of future infectious diseases in society. Environmental Research, vol. 197, June, n. 111099, https://doi.org/10.1016/j.envres.2021.111099

 Askitas, N., Tatsiramos, K., Verheyden, B. 2021. Estimating worldwide effects of non-pharmaceutical interventions on COVID-19 incidence and population mobility patterns using a multiple-event study (Open Access)(2021) Scientific Reports, 11 (1), art. no. 1972.

 Coccia M. (2020). Factors determining the diffusion of COVID-19 and suggested strategy to prevent future accelerated viral infectivity similar to COVID. The Science of the total environment, 729, 138474. https://doi.org/10.1016/j.scitotenv.2020.138474

 Jamison JC, Bundy D, Jamison DT, Spitz J, Verguet S (2021) Comparing the impact on COVID-19 mortality of self-imposed behavior change and of government regulations across 13 countries. Health Serv Res 56(5):874–884. https:// doi. org/ 10. 1111/ 1475- 6773. 13688

 Bontempi E., Coccia M., Vergalli S., Zanoletti A. 2021. Can commercial trade represent the main indicator of the COVID-19 diffusion due to human-to-human interactions? A comparative analysis between Italy, France, and Spain, Environmental Research, vol. 201, Article number 111529, https://doi.org/10.1016/j.envres.2021.111529

 Khandia R, Singhal S, Alqahtani T, Kamal MA, El-Shall NA, Nainu F, Desingu PA, Dhama K (2022) Emergence of SARS-CoV-2 Omicron (B.1.1.529) variant, salient features, high global health concerns and strategies to counter it amid ongoing COVID-19 pandemic. Environ Res 209:112816. https:// doi. org/ 10. 1016/j.envres. 2022. 112816

 Coccia M. 2022. COVID-19 pandemic over 2020 (with lockdowns) and 2021 (with vaccinations): similar effects for seasonality and environmental factors. Environmental Research, Volume 208, 15 May 2022, n. 112711. https://doi.org/10.1016/j.envres.2022.112711

 Diao, Y., Kodera, S., Anzai, D., (...), Rashed, E.A., Hirata, A.    2021. Influence of population density, temperature, and absolute humidity on spread and decay durations of COVID-19: A comparative study of scenarios in China, England, Germany, and Japan, One Health, 12,100203

 Coccia M. 2021. Pandemic Prevention: Lessons from COVID-19. Encyclopedia, vol. 1, n. 2, pp. 433-444. doi: 10.3390/encyclopedia1020036

 Mahmoudi J, Xiong C (2022) How social distancing, mobility, and preventive policies affect COVID-19 outcomes: big data-driven evidence from the District of Columbia-Maryland-Virginia (DMV) megaregion. PLoS One 17(2):e0263820. https:// doi. org/ 10. 1371/journ al. pone. 02638 20

 Coccia M. 2021. High health expenditures and low exposure of population to air pollution as critical factors that can reduce fatality rate in COVID-19 pandemic crisis: a global analysis. Environmental Research, vol. 199, Article number 111339, https://doi.org/10.1016/j.envres.2021.111339

 Qiu Z, Cao Z, Zou M, (...), Wang D, Du X (2022) The effectiveness of governmental nonpharmaceutical interventions against COVID-19 at controlling seasonal influenza transmission: an ecological study. BMC Infect Dis 22(1):331

 Coccia M. 2023. Effects of strict containment policies on COVID-19 pandemic crisis: lessons to cope with next pandemic impacts. Environmental science and pollution research international, 30(1), 2020–2028. https://doi.org/10.1007/s11356-022-22024-w

 Wieland T (2020) A phenomenological approach to assessing the effectiveness of COVID-19 related nonpharmaceutical interventions in Germany. Saf Sci 131:104924

 Coccia M. 2022. COVID-19 Vaccination is not a Sufficient Public Policy to face Crisis Management of next Pandemic Threats. Public Organization Review, https://doi.org/10.1007/s11115-022-00661-6

 Wood SN (2021) Inferring UK COVID-19 fatal infection trajectories from daily mortality data: were infections already in decline before the UK lockdowns? Biometrics. https://doi.org/10.1111/biom.13462.doi:10.1111/biom.13462

 Coccia M. 2022. Improving preparedness for next pandemics: Max level of COVID-19 vaccinations without social impositions to design effective health policy and avoid flawed democracies. Environmental Research, vol. 213,  n. 113566. https://doi.org/10.1016/j.envres.2022.113566

 Zhu P, Tan X (2022) Evaluating the effectiveness of Hong Kong’s border restriction policy in reducing COVID-19 infections. BMC Public Health 22(1):803

Is the work clearly and accurately presented and does it cite the current literature?

Partly

If applicable, is the statistical analysis and its interpretation appropriate?

Yes

Are all the source data underlying the results available to ensure full reproducibility?

Partly

Is the study design appropriate and is the work technically sound?

Partly

Are the conclusions drawn adequately supported by the results?

Partly

Are sufficient details of methods and analysis provided to allow replication by others?

Partly

Reviewer Expertise:

COVID-19 pandemic crisis, crisis management, pandemic prevention, Infectious diseases, epidemiology

I confirm that I have read this submission and believe that I have an appropriate level of expertise to confirm that it is of an acceptable scientific standard, however I have significant reservations, as outlined above.

## References

[1] : Effective Contact Tracing System Minimizes COVID-19 Related Infections and Deaths: Policy Lessons to Reduce the Impact of Future Pandemic Diseases. *Journal of Public Administration and Governance* .2022;12(3) : 10.5296/jpag.v12i3.19834 10.5296/jpag.v12i3.19834

[2] : What the COVID-19 pandemic reveals about science, policy and society. *Interface Focus* .2021;11(6) : 10.1098/rsfs.2021.0022 20210022 10.1098/rsfs.2021.0022 34956594 PMC8504882

[3] : Sources, diffusion and prediction in COVID-19 pandemic: lessons learned to face next health emergency. *AIMS Public Health* .2023;10(1) : 10.3934/publichealth.2023012 145-168 10.3934/publichealth.2023012 37063362 PMC10091135

[4] : Successful Government Responses to the Pandemic. *International Journal of E-Planning Research* .2021;10(2) : 10.4018/IJEPR.20210401.oa1 1-17 10.4018/IJEPR.20210401.oa1

[5] : Preparedness of countries to face COVID-19 pandemic crisis: Strategic positioning and factors supporting effective strategies of prevention of pandemic threats. *Environ Res* .2022;203: 10.1016/j.envres.2021.111678 111678 10.1016/j.envres.2021.111678 34280421 PMC8284056

[6] : SARS-CoV-2 and other pathogenic microorganisms in the environment. *Environmental Research* .2021;201: 10.1016/j.envres.2021.111606 10.1016/j.envres.2021.111606 PMC845933434181924

[7] : Covid-19 Lockdown Cost/Benefits: A Critical Assessment of the Literature. *International Journal of the Economics of Business* .2022;29(1) : 10.1080/13571516.2021.1976051 1-32 10.1080/13571516.2021.1976051

[8] : The impact of first and second wave of the COVID-19 pandemic in society: comparative analysis to support control measures to cope with negative effects of future infectious diseases. *Environ Res* .2021;197: 10.1016/j.envres.2021.111099 111099 10.1016/j.envres.2021.111099 33819476 PMC8017951

[9] : Estimating worldwide effects of non-pharmaceutical interventions on COVID-19 incidence and population mobility patterns using a multiple-event study. *Sci Rep* .2021;11(1) : 10.1038/s41598-021-81442-x 1972 10.1038/s41598-021-81442-x 33479325 PMC7820317

[10] : Factors determining the diffusion of COVID-19 and suggested strategy to prevent future accelerated viral infectivity similar to COVID. *Sci Total Environ* .2020;729: 10.1016/j.scitotenv.2020.138474 138474 10.1016/j.scitotenv.2020.138474 32498152 PMC7169901

[11] : Comparing the impact on COVID-19 mortality of self-imposed behavior change and of government regulations across 13 countries. *Health Serv Res* .2021;56(5) : 10.1111/1475-6773.13688 874-884 10.1111/1475-6773.13688 34182593 PMC8441808

[12] : Can commercial trade represent the main indicator of the COVID-19 diffusion due to human-to-human interactions? A comparative analysis between Italy, France, and Spain. *Environ Res* .2021;201: 10.1016/j.envres.2021.111529 111529 10.1016/j.envres.2021.111529 34147467 PMC8459318

[13] : Emergence of SARS-CoV-2 Omicron (B.1.1.529) variant, salient features, high global health concerns and strategies to counter it amid ongoing COVID-19 pandemic. *Environmental Research* .2022;209: 10.1016/j.envres.2022.112816 10.1016/j.envres.2022.112816 PMC879878835093310

[14] : COVID-19 pandemic over 2020 (withlockdowns) and 2021 (with vaccinations): similar effects for seasonality and environmental factors. *Environ Res* .2022;208: 10.1016/j.envres.2022.112711 112711 10.1016/j.envres.2022.112711 35033552 PMC8757643

[15] : Influence of population density, temperature, and absolute humidity on spread and decay durations of COVID-19: A comparative study of scenarios in China, England, Germany, and Japan. *One Health* .2021;12: 10.1016/j.onehlt.2020.100203 100203 10.1016/j.onehlt.2020.100203 33344745 PMC7736723

[16] : Pandemic Prevention: Lessons from COVID-19. *Encyclopedia* .2021;1(2) : 10.3390/encyclopedia1020036 433-444 10.3390/encyclopedia1020036

[17] : Proteomic analysis of metabolic pathways supports chloroplast-mitochondria cross-talk in a Cu-limited diatom. *Plant Direct* .2022;6(1) : 10.1002/pld3.376 e376 10.1002/pld3.376 35079683 PMC8777261

[18] : High health expenditures and low exposure of population to air pollution as critical factors that can reduce fatality rate in COVID-19 pandemic crisis: a global analysis. *Environ Res* .2021;199: 10.1016/j.envres.2021.111339 111339 10.1016/j.envres.2021.111339 34029545 PMC8139437

[19] : The effectiveness of governmental nonpharmaceutical interventions against COVID-19 at controlling seasonal influenza transmission: an ecological study. *BMC Infect Dis* .2022;22(1) : 10.1186/s12879-022-07317-2 331 10.1186/s12879-022-07317-2 35379168 PMC8977560

[20] : Effects of strict containment policies on COVID-19 pandemic crisis: lessons to cope with next pandemic impacts. *Environ Sci Pollut Res Int* .2023;30(1) : 10.1007/s11356-022-22024-w 2020-2028 10.1007/s11356-022-22024-w 35925462 PMC9362501

[21] : A phenomenological approach to assessing the effectiveness of COVID-19 related nonpharmaceutical interventions in Germany. *Saf Sci* .2020;131: 10.1016/j.ssci.2020.104924 104924 10.1016/j.ssci.2020.104924 32834516 PMC7373035

[22] : COVID-19 Vaccination is not a Sufficient Public Policy to face Crisis Management of next Pandemic Threats. *Public Organization Review* .2023;23(4) : 10.1007/s11115-022-00661-6 1353-1367 10.1007/s11115-022-00661-6

[23] : Inferring UK COVID-19 fatal infection trajectories from daily mortality data: Were infections already in decline before the UK lockdowns?. *Biometrics* .2022;78(3) : 10.1111/biom.13462 1127-1140 10.1111/biom.13462 33783826 PMC8251436

[24] : Improving preparedness for next pandemics: Max level of COVID-19 vaccinations without social impositions to design effective health policy and avoid flawed democracies. *Environ Res* .2022;213: 10.1016/j.envres.2022.113566 113566 10.1016/j.envres.2022.113566 35660409 PMC9155186

[25] : Evaluating the effectiveness of Hong Kong's border restriction policy in reducing COVID-19 infections. *BMC Public Health* .2022;22(1) : 10.1186/s12889-022-13234-5 803 10.1186/s12889-022-13234-5 35449094 PMC9023047
